# Pharmacokinetics, Safety, and Tolerability of NPC‐21, an Anti‐Cytomegalovirus Monoclonal Antibody, in Healthy Japanese and White Adult Men: A Randomized, Placebo‐Controlled, First‐in‐Human Phase 1 Study

**DOI:** 10.1002/cpdd.1065

**Published:** 2022-01-05

**Authors:** Kenichi Furihata, Izumi Hamada, Takuro Niwa, Tatsuya Watanabe, Sachiko Ezoe

**Affiliations:** ^1^ P‐one Clinic Keikokai Medical Corporation Tokyo Japan; ^2^ Research & Development Division Nobelpharma Co. Ltd. Tokyo Japan; ^3^ Department of Space Infection Control, Graduate School of Medicine, Division of Health Science Osaka University Osaka Japan; ^4^ Medical Center for Translational Research Osaka University Hospital Osaka Japan

**Keywords:** antigenic domain 1, first‐in‐human phase 1 study, human cytomegalovirus, monoclonal antibody, NPC‐21, pharmacokinetic

## Abstract

NPC‐21 (EV2038) is a fully human monoclonal antibody that targets the antigenic domain 1 of glycoprotein B on the human cytomegalovirus (hCMV) envelope. NPC‐21 has been shown to have broadly neutralizing activity and to inhibit cell‐to‐cell transmission of hCMV in preclinical studies. It is currently in development for the prophylactic or preemptive treatment of hCMV in patients receiving a solid‐organ transplant or hematopoietic stem cell transplant. A first‐in‐human phase 1 study was conducted to assess the pharmacokinetics, safety, and tolerability of NPC‐21 in healthy adult men. Forty participants (Japanese, n = 32; White, n = 8) were randomly assigned to receive a single intravenous dose of NPC‐21 1, 3, 10, or 20 mg/kg or placebo. Six Japanese participants were included in each dose group and six White participants received a 10‐mg/kg dose. The placebo group included 8 Japanese participants and 2 White participants. All 40 participants completed the study. Serum concentration, maximum serum concentration, area under the plasma concentration–time curve from time 0 to the last measurable concentration, and area under the plasma concentration–time curve from time 0 to infinity increased dose dependently; dose proportionality was linear. NPC‐21 demonstrated a biphasic elimination pattern, with an estimated half‐life between 612 and 790 hours. NPC‐21 was safe and well tolerated up to 20 mg/kg. All adverse events were mild, and none led to treatment discontinuation or were considered related to the study drug. There were no differences in pharmacokinetics or safety between Japanese and White participants. These results support further investigation of NPC‐21.

Human cytomegalovirus (hCMV) is a β‐herpesvirus (type 5). Infection with hCMV is common, with an estimated 50% to 90% incidence of infection globally.^1^ Congenital hCMV infection is usually asymptomatic but can result in serious sequelae, such as hearing loss, in about half of the 10% to 15% of infants who are symptomatic.^2^, ^3^, ^4^ Primary infection with hCMV during puberty or later often manifests with infectious mononucleosis–like symptoms.^5^ Whereas healthy individuals usually develop no or only mild symptoms, individuals with reduced immunity, such as those with acquired immunodeficiency or malignant tumors, or those receiving a solid‐organ transplant (SOT) or hematopoietic stem cell transplant (HSCT) develop significant symptoms that result in a poor prognosis.^6^, ^7^ hCMV infection is considered lifelong; although the virus goes into latency after primary infection, episodes of reactivation may occur, particularly in those who are immunocompromised.

SOT recipients with hCMV infection have an increased risk of morbidity and mortality within 6 months of transplantation.^8^, ^9^ Additionally, they may experience chronic inflammation and increased risk of allograft injury or rejection^10^, ^11^ or develop new opportunistic infections.^12^ In the absence of prophylactic or preemptive treatment, 40% to 60% of patients receiving SOT develop symptomatic hCMV infections, with the most severe infections and highest incidence occurring when organs from hCMV‐seropositive individuals are transplanted into hCMV‐seronegative recipients.^7^ HSCT recipients with hCMV may develop CMV pneumonia, which has a high fatality rate even with treatment, or organ damage due to infection.^13^ Indirect effects of infection for patients receiving HSCT include the possibility of graft failure, immunosuppression, or the development of new infections.^1^ For HSCT recipients, the risk is highest among hCMV‐seropositive recipients, regardless of the serostatus of the donor. Without preemptive treatment, ≈80% of hCMV‐seropositive HSCT recipients will experience hCMV infection.^13^

In clinical practice, prophylactic or preemptive treatment is given to SOT and HSCT recipients.^7^, ^8^, ^13^ Ganciclovir (GCV) is the first‐line antiviral drug used for treatment and, recently, letermovir was approved for use in HSCT recipients in the United States, Canada, Europe, and Japan.^14^ The use of GCV and other antiviral drugs may lead to serious adverse drug reactions, such as bone marrow depression, renal disorders, or fertility issues.^15^ Whereas letermovir is not associated with renal or hematopoietic adverse effects, dose reductions are recommended when treating patients who are receiving cyclosporine.^16^, ^17^ Additionally, treatment with GCV or letermovir may promote the development of GCV or letermovir‐resistant hCMV strains.^16^, ^18^

Outside of Japan, high‐titer CMV–immune globulin intravenous (IVCytoGam) is available and can be used alone or in combination with antiviral drugs. Because CMV–immune globulin IV is a blood product, there are concerns related to the transmission of blood‐borne viruses and lot‐to‐lot variations in quality. Therefore, development of a nonblood product with antibody activity would be beneficial for the control of hCMV infection in susceptible individuals.

NPC‐21 (EV2038) is a fully human immunoglobin G1λ monoclonal antibody isolated from Epstein‐Barr virus–transformed peripheral B cells of a healthy donor. NPC‐21 targets antigenic domain 1 (AD‐1) of glycoprotein B (gB), which is the dominant antigen on the hCMV envelope.^19^ AD‐1 is highly conserved among clinical hCMV strains and is essential for the function of gB^20^, ^21^, ^22^; gB is necessary for the entry, fusion, and cell‐to‐cell spread of hCMV.^23^ In preclinical in vitro studies using plaque reduction assays, NPC‐21 has demonstrated broadly neutralizing activity against a wide range of hCMV strains, including 4 laboratory strains (AD169, Towne, Davis, Merlin) and 42 Japanese clinical isolates, which included GCV‐resistant isolates (Nobelpharma Co., Ltd., Tokyo, Japan; data on file). In MRC‐5 human embryonic fibroblast cells and human adult retinal pigment epithelial cells, the 50% inhibitory concentration of NPC‐21 ranged from 0.013 to 0.105 μg/mL and the 90% inhibitory concentration ranged from 0.208 to 1.026 μg/mL. NPC‐21 also inhibits cell‐to‐cell infection by hCMV clinical isolates (n = 8) in adult retinal pigment epithelial cells (90% inhibitory concentration, 13‐19 μg/mL).

As a fully human antibody, NPC‐21 has the primary pharmacologic action of neutralizing exogenous antigens. In a 4‐week repeated‐dose toxicity study conducted in cynomolgus monkeys (once weekly intravenous [IV] administration), the dosage level at which no adverse effects were observed was 50 mg/kg (Nobelpharma Co., Ltd.; data on file).

The purpose of this randomized, placebo‐controlled, first‐in‐human phase 1 study was to evaluate the pharmacokinetics, safety, and tolerability of a single IV administration of NPC‐21 in both Japanese and White healthy men participants in Japan.

## Methods

The study protocol was approved by the institutional review board of the study site (P‐One Clinic, Keikokai Medical Corporation, Tokyo, Japan). All participants provided written informed consent. The study was conducted in compliance with ethical principles that have their origins in the Declaration of Helsinki (1964) and its revised version; the standards specified in Paragraph 3, Article 14, and Article 80‐2 of the Pharmaceuticals and Medical Devices Act; relevant laws and regulations, including the Ordinance on Good Clinical Practice (Ordinance of the Ministry of Health, Labour and Welfare No. 28 dated 27 March 1997); and the study protocol.

### Participants

Study participants were healthy Japanese or White men aged 20 to <45 years, with a body mass index of 18.5 to <25.0 kg/m^2^ for Japanese participants or 18.5 to <27.5 kg/m^2^ for White participants, who had not used other drugs or related products within 2 weeks or nicotine within 3 months before receiving the study treatment, did not have any significant disease or abnormal screening results, and did not have a history of serious drug allergy. All participants provided written informed consent.

### Study Design

This was a randomized, placebo‐controlled, double‐blind, dose‐ascending, phase 1 study to determine the pharmacokinetics, safety, and tolerability of a single IV injection of NPC‐21 in Japanese and White healthy adult male participants in Japan. A randomization schedule was created for each step using a random number generator function. The individual responsible for investigational product allocation created the randomization schedule. All operations related to randomization were conducted in a locked location accessible only to the individual responsible for investigational product allocation, their assistant, and unblinded dispensing staff.

The study design is shown in Figure S1. This study was conducted in 5 steps and comprised 4 dose groups. Japanese participants received a single IV dose of 1, 3, 10, or 20 mg/kg, and White participants received a single IV dose of 10 mg/kg NPC‐21. Pharmacokinetics and safety were compared between the White group and the Japanese group. For each of the 5 steps, 6 participants received NPC‐21, and 2 received placebo. Step 1 was conducted in 2 parts, as this was the first time NPC‐21 had been administered to humans. For the first part, 1 participant each received NPC‐21 (1 mg/kg) or placebo; after the safety evaluation of these first 2 participants at day 8, an additional 5 participants received NPC‐21 (1 mg/kg) and 1 participant received placebo if no safety concerns had been determined by the study investigator. For steps 2 through 4, dosing was initiated on day 29 of the previous step if the medical expert and study investigator were able to determine that there were no safety concerns for the previous dose. If safety could not be verified according to the step transition criteria or dose escalation was deemed to be inappropriate, then the study would be discontinued without advancing to the next step. The step transition criteria dictated that if any of the following conditions were met, it would be considered that safety could not be verified: a severe or serious adverse event (AE) for which a causal relationship with NPC‐21 could not be ruled out, or any AE in which a dose increase was deemed to significantly affect the health of participants. Steps 3‐1 and 3‐2 (10 mg/kg in Japanese or White participants, respectively) were conducted in parallel. The placebo group included 8 Japanese participants and 2 White participants.

NPC‐21 (diluted in sterile saline) or placebo (sterile saline) was administered by IV infusion (5 mL/min; 60 minutes) in a volume of ≈300 mL. Participants were hospitalized from day −1 to day 4, and outpatient visits were conducted on days 8, 15, 22, 29, 43, 57, and 85. A randomized, placebo‐controlled, double‐blind study design was adopted to minimize bias.

### Pharmacokinetic Assessments

Pharmacokinetic outcomes included serum concentrations over time and pharmacokinetic parameters, including area under the plasma concentration–time curve from time 0 to the last measurable concentration (AUC_0‐t_), AUC from time 0 to infinity (AUC_0‐∞_), total body clearance (Cl_B_), maximum serum concentration (C_max_), half‐life (t_1/2_), time to reach maximum serum concentration (t_max_), and volume distribution (V_d[area]_).

Pharmacokinetic parameters were calculated using serum NPC‐21 concentrations. Blood samples (≈2 mL) were collected from the antebrachial vein at each of the following sampling times: before study drug administration and at 0.5, 1, 2, 4, 8, 12, 24, 36, 48, and 72 hours, and days 8, 15, 22, 29, 43, 57, and 85 after administration. After separation, serum was stored at −70˚C until analysis. Separate fully validated electrochemiluminescence‐based assays were used to determine serum NPC‐21 concentration and to detect anti–NPC‐21 antibodies.

Serum NPC‐21 concentrations were determined by sandwich enzyme‐linked immunosorbent assay^24^ using an anti‐idiotype monoclonal antibody specific for NPC‐21 (Evec, Inc., Sapporo, Japan; capture antibody), a biotin‐labeled anti‐idiotype monoclonal antibody (different from the capture antibody) specific for NPC‐21 (Evec, Inc.; detection antibody; biotin labeling by Shin Nippon Biomedical Laboratories, Ltd., Pharmacokinetics and Bioanalysis Center, Wakayama, Japan) and SULFO‐TAG labeled streptavidin (Meso Scale Diagnostics, LLC, Rockville, Maryland). NPC‐21 was used as the reference standard and pooled blank serum was used as a negative control (diluted 100‐fold in assay buffer). Serum samples were prepared by diluting the study serum sample into assay buffer (minimum required dilution: 100‐fold; dilution linearity, up to 12 500‐fold with pooled blank serum). Plates were read using an electrochemiluminescence plate reader (MESO QuickPlex SQ120; Meso Scale Diagnostics, LLC). The calibration standard curve was fitted through 8 calibration standards (25‐3200 ng/mL) using a 4‐parameter logistic curve. The lower limit of quantification for this assay was 25 ng/mL. Within‐ and between‐day accuracy and precision were confirmed using quality control samples of diluted NPC‐21 (3200, 2400, 300, 75, and 25 ng/mL). Precision (coefficient of variation) was limited to ≤20.0%, and accuracy was limited to within ±20%.

### Safety Assessments

AEs (throughout the study, starting at the time of administration), laboratory tests (screening, day −1, and days 2 [24 hours], 4 [72 hours], 8, 15, 22, 29, 43, 57, and 85 after administration), vital signs (screening, day −1, and days 1 [before administration and 0.5, 1, 4, and 12 hours], 2 [24 hours], 3 [48 hours], 4 [72 hours], 8, 15, 22, 29, 43, 57, and 85 after administration), resting 12‐lead electrocardiogram (screening, day −1, and days 1 [4 hours], 2 [24 hours], 4 [72 hours], 8, 15, 29, 57, and 85 after administration), and anti–NPC‐21 antibodies (day 1 [before administration] and days 15, 29, 57, and 85 [after administration]) were monitored to evaluate safety. AEs were coded according to System Organ Class and Preferred Terms using the Medical Dictionary for Regulatory Activities version 21.1.

A bridging format with biotin‐labeled NPC‐21 (capture antibody) and ruthenium‐labeled NPC‐21 (detection antibody) was used to detect serum anti–NPC‐21 antibodies.^25^ An anti‐idiotype monoclonal antibody specific for NPC‐21 (Evec, Inc.) was used as a positive control (80 000 ng/mL and 4000 ng/mL diluted in pooled blank serum), and pooled blank serum was used as a negative control (diluted 16‐fold in dilution buffer). Serum samples were prepared by 16‐fold dilution in dilution buffer. Plates were read using an electrochemiluminescence plate reader (MESO QuickPlex SQ120; Meso Scale Diagnostics, LLC). For initial screening, measured values below the cut‐point value (mean of measured values of negative control samples × normalization factor [1.22]) were considered negative for anti–NPC‐21 antibodies, and values equal to or greater than the cut‐point value were considered positive. The allowed coefficient of variation between sample duplicates was limited to ≤20.0%. If a sample was considered positive in the screening assay, a confirmatory assay was performed in which samples were or were not spiked with antigen (NPC‐21) followed by incubation for 30 ± 5 minutes at 25˚C. Percent inhibition was calculated as follows: (1 − [measured valued of sample spiked with antigen / measured value of sample without antigen]) × 100. A sample was considered positive if percent inhibition was greater than or equal to the specificity cut point (24.0%). Titers for positive samples were determined in a second confirmatory assay.

### Statistical Analysis

Because this was an exploratory study, no formal statistical analyses were used to determine sample size. The number of participants needed to evaluate the tolerability, safety, and pharmacokinetics of NPC‐21 was estimated to be 6 for the NPC‐21 group and 2 for the placebo group at each dose level (total of 40 study participants). The pharmacokinetic population consisted of all participants receiving NPC‐21 or placebo and having serum concentration obtained at 1 or more time points after the start of study treatment. The safety population consisted of all participants receiving NPC‐21 or placebo.

Summary statistics were calculated for continuous data, and frequency distribution was calculated for categorical data. Pharmacokinetic parameters (C_max_, t_max_, AUC_0‐t_, AUC_0‐∞_, t_1/2_, Cl_B_, and V_d[area]_) were calculated from serum NPC‐21 concentrations using noncompartmental analysis. Dose linearity of pharmacokinetic parameters (AUC_0–t_, AUC_0‐∞_, and C_max_) was calculated by both linear regression analysis and a power model (using a log‐transformed linear regression analysis). For the linear regression analysis, the following equation was used: *Y* = α + β*X*, where *X* is the dose and *Y* is the pharmacokinetic parameter (C_max_, AUC_0–t_, or AUC_0‐∞_). The dose linearity was examined by calculating an estimate of α, CI, and the *P* value. For the log‐transformed linear regression analysis, the following equation was used: *ln*(α) + β*ln*(*X*), where *X* is the dose and *Y* is the pharmacokinetic parameter (C_max_, AUC_0–t_, or AUC_0‐∞_). Dose linearity was examined by calculating an estimate of β, CI, and the *P* value.

A 2‐sided significance level of ≤.1 was set for the assessment of pharmacokinetic parameters, and ≤.05 was set for all other parameters. All statistical analyses were performed using Phoenix WinNonlin 8.1 (Certara, LP, Princeton, New Jersey) and SAS version 9.4 (SAS Institute Inc., Cary, North Carolina).

## Results

### Participants

Participant disposition is shown in Figure S2. In total, 40 participants were enrolled, received the study drug, and completed the study at a single center in Japan. The study was conducted between July 26, 2018, and March 8, 2019; none of the participants withdrew or discontinued the study.

The background characteristics of the study participants are shown in Table 1. Participant characteristics were similar between the dosing groups and between the Japanese and White participants; approximately half of the study population had anti‐CMV immunoglobulin G antibodies. Importantly, the mean (standard deviation) body mass index of the Japanese and White participants who received 10 mg/kg NPC‐21 was similar (22.0 [1.9] and 24.1 [2.3], respectively). All 40 participants were included in both the safety and pharmacokinetic populations.

**Table 1:** Participant Background Characteristics

	NPC‐21					
	1 mg/kg	3 mg/kg	10 mg/kg Japanese	10 mg/kg White	20 mg/kg	Placebo
	n = 6	n = 6	n = 6	n = 6	n = 6	n = 10
Age, y	37.7 (6.4)	24.5 (4.4)	32.0 (9.2)	32.7 (8.7)	31.5 (10.1)	31.5 (8.4)
Height, cm	170.8 (4.9)	171.9 (4.0)	171.3 (6.0)	179.0 (6.9)	169.2 (4.1)	176.9 (4.8)
Weight, kg	64.9 (7.5)	61.9 (8.9)	64.5 (6.2)	77.1 (9.0)	62.5 (4.5)	69.2 (7.5)
BMI, kg/m^2^	22.2 (1.6)	20.9 (2.0)	22.0 (1.9)	24.1 (2.3)	21.9 (1.8)	22.1 (1.7)
CMV IgG, n (%)						
Negative	3 (50.0)	4 (66.7)	2 (33.3)	2 (33.3)	2 (33.3)	5 (50.0)
Positive	3 (50.0)	2 (33.3)	4 (66.7)	4 (66.7)	4 (66.7)	5 (50.0)
CMV IgM, n (%)						
Negative	6 (100.0)	6 (100.0)	6 (100.0)	6 (100.0)	5 (83.3)	9 (90.0)
Positive	0 (0.0)	0 (0.0)	0 (0.0)	0 (0.0)	1 (16.7)	1 (10.0)

BMI, body mass index; CMV, cytomegalovirus; Ig, immunoglobin.

Data are mean (standard deviation).

### Pharmacokinetic Outcomes

**Figure 1:**
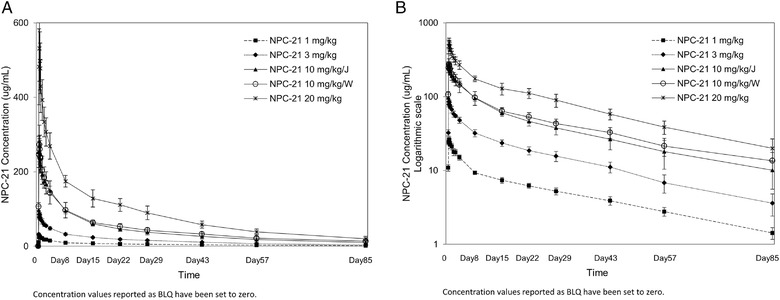
Changes over time in serum NPC‐21 concentrations (mean ± standard deviation). (A) Data shown using a linear scale. (B) Data shown using a logarithmic scale. BLQ, below the limit of quantification; J, Japanese; W, White.

Changes in serum concentration of NPC‐21 over time are shown in Figure 1. Serum NPC‐21 concentrations increased with increased dose, and changes over time were similar between Japanese and White participants who were given a single dose of 10 mg/kg NPC‐21.

**Figure 2:**
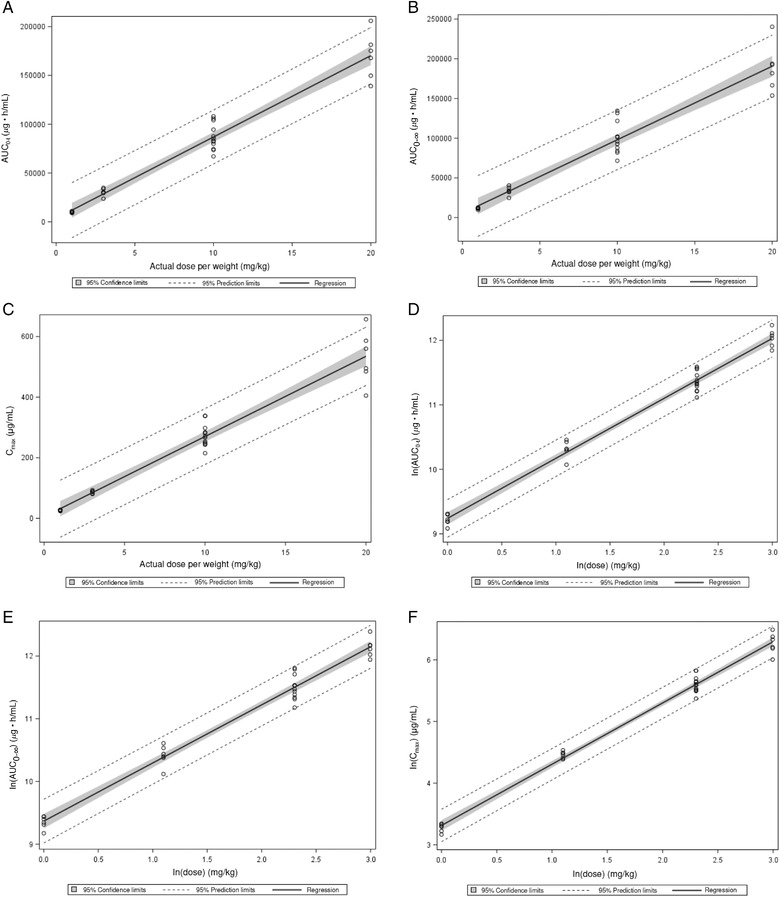
Regression for dose proportionality by linear regression analysis ([A] AUC_0‐t_, [B] AUC_0‐∞_, [C] C_max_) and by power model analysis ([D] AUC_0‐t_, [E] AUC_0‐∞_, [F] C_max_). AUC_0‐t_, area under the plasma concentration–time curve from time 0 to the last measurable concentration; AUC_0‐∞_, area under the plasma concentration–time curve from time 0 to infinity; C_max_, maximum serum concentration.

Summary statistics for each of the pharmacokinetic parameters by dose group are shown in Table 2. T_max_ was lowest in the 20 mg/kg group (2 hours) and greatest in the 3 mg/kg group (3.67 hours). The t_1/2_ was estimated to be between 612 and 790 hours and the elimination pattern was considered biphasic. In the comparison of Japanese and White participants, the mean AUC_0‐t_ was 82 700 μg • h/mL vs 91 600 μg • h/mL, and the mean C_max_ was 273 μg/mL vs 274 μg/mL, respectively. C_max_, AUC_0‐t_, and AUC_0‐∞_ increased in a dose‐dependent fashion; dose proportionality was demonstrated by a linear regression analysis and a power model analysis. Linear regression analysis showed an intercept (95%CI) for α of 3580 (−4617.99 to 11 786.22) for AUC_0‐t_ (Figure 2A), 5420 (−5714.30 to 16 553.65) for AUC_0‐∞_ (Figure 2B), and 5.35 (−22.05 to 32.75) for C_max_ (Figure 2C), thus meeting the requirement that 0 is included in the 95%CI of α.^26^ In the power model analysis of AUC_0‐t_ (Figure 2D), AUC_0‐∞_ (Figure 2E), and C_max_ (Figure 2F), the estimated β (95%CI) was 0.930 (0.88‐0.98), 0.927 (0.87‐0.98), and 0.993 (0.95‐1.04), respectively. Thus, the point estimates of β were included in the range between 0.85 and 1.15, and the 95%CIs in the range between 0.7 and 1.3, which statistically verified linearity.^26^ There were no significant differences between dose levels for t_max_, t_1/2_, Cl_B_, and V_d(area)_.

**Table 2:** Pharmacokinetic Parameters Following Intravenous Administration of NPC‐21

NPC‐21					
	1 mg/kg	3 mg/kg	10 mg/kg, Japanese	10 mg/kg, White	20 mg/kg
	n = 6	n = 6	n = 6	n = 6	n = 6
AUC_0‐t_, μg • h/mL	10 100 (826)	30 300 (3920)	82 700 (13 400) 90%CI (71 683‐93 723)	91 600 (13 000) 90%CI (80 946‐102281)	170 000 (23 800)
AUC_0‐∞_, μg • h/mL	11 600 (1120)	33 700 (5360)	93 600 (20 500) 90%CI (76 754‐110 539)	108 000 (17 800) 90%CI (92 889‐122 229)	188 000 (30 000)
C_max_, μg/mL	26.5 (1.78)	86.6 (4.74)	273 (18.9) 90%CI (257‐288)	274 (52.0) 90%CI (232‐317)	531 (88.4)
t_max_, μg/mL	2.33 (0.82)	3.67 (2.34)	3.00 (2.45) 90%CI (0.98‐5.02)	2.67 (1.03) 90%CI (1.82‐3.52)	2.00 (0)
t_1/2_, h	707 (82.0)	612 (95.3)	706 (133) 90%CI (597.13‐815.68)	790 (142) 90%CI (673.28‐907.53)	620 (76.2)
Cl_B_, mL/h/kg	0.0873 (0.0092)	0.0912 (0.0163)	0.111 (0.0211) 90%CI (0.0932‐0.1280)	0.0951 (0.0156) 90%CI (0.0823‐0.1079)	0.108 (0.0161)
V_d(area)_, mL/kg	88.4 (8.69)	78.9 (4.41)	110 (11.0) 90%CI (100.85‐118.89)	107 (19.3) 90%CI (91.31‐123.01)	96.6 (16.6)

AUC_0‐t_, area under the plasma concentration–time curve from time 0 to the last measurable concentration; AUC_0‐∞_, area under the plasma concentration–time curve from time 0 to infinity; Cl_B_, total body clearance; C_max_, maximum serum concentration; t_1/2_, half‐life; t_max_, time to reach maximum serum concentration; V_d(area)_, volume of distribution based on the AUC.

Data are mean (standard deviation) unless otherwise stated.

### Safety Outcomes

AEs are listed in Table 3. A total of 23 AEs were reported in 16 participants; all were nonserious and mild. Nasopharyngitis was the most common AE, with 11 occurrences in 10 participants. No AEs led to discontinuation, and none were judged by the investigator to have a causal relationship with the study drug. NPC‐21 was well tolerated, and there were no safety issues up to the maximum dose of 20 mg/kg, and no relationship between the frequency of AEs and dose. There was no difference in the incidence of AEs between Japanese and White participants at the same dose (10 mg/kg).

**Table 3:** Adverse Events

System Organ Class	1 mg/kg	3 mg/kg	10 mg/kg, Japanese	10 mg/kg, White	20 mg/kg	Placebo
Preferred Term	n = 6	n = 6	n = 6	n = 6	n = 6	n = 10
Any adverse event	2 (33.3); 4	3 (50.0); 3	3 (50.0); 4	3 (50.0); 4	2 (33.3); 2	3 (30.0); 6
Gastrointestinal disorders	1 (16.7); 1	0 (0.0); 0	0 (0.0); 0	0 (0.0); 0	0 (0.0); 0	0 (0.0); 0
Dental caries	1 (16.7); 1	0 (0.0); 0	0 (0.0); 0	0 (0.0); 0	0 (0.0); 0	0 (0.0); 0
Infections and infestations	0 (0.0); 0	2 (33.3); 2	3 (50.0); 3	3 (50.0); 3	1 (16.7); 1	1 (10.0); 2
Nasopharyngitis	0 (0.0); 0	2 (33.3); 2	3 (50.0); 3	3 (50.0); 3	1 (16.7); 1	1 (10.0); 2
Injury, poisoning, and procedural complications	1 (16.7); 1	0 (0.0); 0	0 (0.0); 0	0 (0.0); 0	0 (0.0); 0	0 (0.0); 0
Facial bones fracture	1 (16.7); 1	0 (0.0); 0	0 (0.0); 0	0 (0.0); 0	0 (0.0); 0	0 (0.0); 0
Laboratory investigations	1 (16.7); 1	0 (0.0); 0	1 (16.7); 1	1 (16.7); 1	0 (0.0); 0	2 (20.0); 4
ALT increased	0 (0.0); 0	0 (0.0); 0	0 (0.0); 0	0 (0.0); 0	0 (0.0); 0	1 (10.0); 1
AST increased	0 (0.0); 0	0 (0.0); 0	0 (0.0); 0	0 (0.0); 0	0 (0.0); 0	1 (10.0); 1
Blood creatine phosphokinase increased	1 (16.7); 1	0 (0.0); 0	0 (0.0); 0	1 (16.7); 1	0 (0.0); 0	1 (10.0); 1
Blood glucose increased	0 (0.0); 0	0 (0.0); 0	1 (16.7); 1	0 (0.0); 0	0 (0.0); 0	0 (0.0); 0
Blood LDH increased	0 (0.0); 0	0 (0.0); 0	0 (0.0); 0	0 (0.0); 0	0 (0.0); 0	1 (10.0); 1
Musculoskeletal and connective tissue disorders	0 (0.0); 0	0 (0.0); 0	0 (0.0); 0	0 (0.0); 0	1 (16.7); 1	0 (0.0); 0
Back pain	0 (0.0); 0	0 (0.0); 0	0 (0.0); 0	0 (0.0); 0	1 (16.7); 1	0 (0.0); 0
Nervous system disorders	0 (0.0); 0	1 (16.7); 1	0 (0.0); 0	0 (0.0); 0	0 (0.0); 0	0 (0.0); 0
Headache	0 (0.0); 0	1 (16.7); 1	0 (0.0); 0	0 (0.0); 0	0 (0.0); 0	0 (0.0); 0
Respiratory, thoracic, and mediastinal disorders	1 (16.7); 1	0 (0.0); 0	0 (0.0); 0	0 (0.0); 0	0 (0.0); 0	0 (0.0); 0
Oropharyngeal pain	1 (16.7); 1	0 (0.0); 0	0 (0.0); 0	0 (0.0); 0	0 (0.0); 0	0 (0.0); 0

ALT, alanine aminotransferase; AST, aspartate aminotransferase; LDH, lactate dehydrogenase.

Data are n (%); total events.

All 6 participants (1 from each group) who were positive for anti–NPC‐21 antibodies had these antibodies before receiving the study drug; therefore, there were no cases of newly developed anti–NPC‐21 antibodies after study drug administration. None of the participants who had anti–NPC‐21 antibodies before study drug administration had increased antibodies after administration.

## Discussion

We report that, at all doses tested, AUC_0‐t_, AUC_0‐∞_, and C_max_ increased in a dose‐dependent manner, while all other pharmacokinetic parameters did not differ significantly by dose (t_max_, t_1/2_, Cl_B_, and V_d[area]_). NPC‐21 was generally safe and well tolerated at all doses, thus confirming the safety and tolerability of the study drug up to 20 mg/mL. All AEs were mild, and there were no serious or severe AEs, no AEs related to the study drug, and no AEs that led to study discontinuation. Finally, there were no differences in the pharmacokinetics or safety observed between Japanese and White participants who received a 10‐mg/kg dose of NPC‐21. The similar pharmacokinetic and safety findings between Japanese and White participants indicate that NPC‐21 clinical studies are appropriate for both Japanese and White patients.

Overall, the pharmacokinetics of NPC‐21 are similar to those reported for other monoclonal antibody drugs. Linear pharmacokinetic behavior is typical for most immunoglobulin G1 antibody drugs and t_1/2_ tends to be around 18 to 21 days.^27^ We report a somewhat longer t_1/2_ for NPC‐21 of 26 to 33 days (612‐790 hours).

Infusion‐related reactions are known to occur with a number of monoclonal antibody drugs. Rates are particularly high (>50%) for rituximab, avelumab, and daratumumab.^28^ Premedication is often used to reduce the risk of such reactions.^29^ Most severe infusion‐related reactions occur during the first or second transfusion.^29^ Therefore, it is encouraging that no allergic or infusion‐related reactions were reported for the present study, and that the patients could be managed without the use of premedication. However, we acknowledge that this study had a small sample size, which precludes extrapolation of these findings to a larger patient population. Larger clinical trials with repeated dosing will be needed to confirm this finding.

There is potential for the development of antidrug antibodies against monoclonal antibody drugs. Antidrug antibodies can reduce the efficacy of monoclonal antibodies through neutralization and alter the pharmacokinetic and pharmacodynamic properties of the drug.^30^ While the present study did identify participants with antidrug antibodies, these antibodies were present before administration of the study drug, and there was no observed effect on the pharmacokinetics of NPC‐21. Neutralizing activity was not evaluated in the present study, so it is unknown whether these antidrug antibodies had neutralizing activity. Future studies are needed to confirm whether antidrug antibodies affect the pharmacokinetics of NPC‐21 and to evaluate the effect of antidrug antibodies on the efficacy of NPC‐21. There is also potential for the development of treatment‐resistant strains of hCMV, as this has been observed for both GCV and letermovir.^16^, ^18^ The present study did not test for NPC‐21–resistant strains, but an ongoing clinical trial of the safety and efficacy of prophylactic NPC‐21 in patients undergoing a high‐risk kidney transplant (NCT04225923) will evaluate whether NPC‐21–resistant strains of hCMV emerge with treatment and whether anti–NPC‐21 antibodies have neutralizing activity.

GCV is used as first‐line prophylactic treatment for SOT recipients and as preemptive treatment for HSCT recipients. However, given the concerns with serious adverse drug reactions (eg, bone marrow depression, renal disorders, and fertility issues) and drug‐drug interactions, both of which may require dose adjustment for hCMV treatment, safer alternatives are needed. There are generally few concerns regarding drug‐drug interactions and dose adjustment for renal dysfunction with antibody drugs.^31^ Coupled with the positive safety data reported in the present study, this leads us to expect a favorable safety profile for NPC‐21. The findings reported herein support the further evaluation of NPC‐21 for treatment of hCMV. A recent review noted the potential importance of anti‐hCMV monoclonal antibodies in the prevention of hCMV infection for high‐risk patients receiving SOT, given their specificity and limited toxicity.^32^ As previously mentioned, a clinical study to determine the efficacy and safety of prophylactic treatment with NPC‐21 in patients who undergo kidney transplant from an hCMV‐seropositive donor is ongoing (NCT04225923).

This study had several limitations. First, only male participants were included, limiting the generalizability of the study findings. Second, only healthy individuals participated, meaning further studies will be needed to assess safety and pharmacokinetics in individuals with reduced organ function. Third, the age of study participants was limited to 20 to <45 years of age; therefore, it is unknown how patients in other age groups may react to NPC‐21. Fourth, although it is anticipated that NPC‐21 will be administered monthly for multiple cycles when used to treat patients, there was no repeated dosing. Finally, as with all phase 1 studies, the number of participants was low. The previously mentioned ongoing phase 2 study will address many of these limitations.

## Conclusions

NPC‐21 was safe and well tolerated up to 20 mg/kg in healthy Japanese men and at the dose of 10 mg/kg in healthy White men. The pharmacokinetic parameters of AUC_0‐t_, AUC_0‐∞_, and C_max_ increased in a dose‐dependent manner, and the t_1/2_ of NPC‐21 was 612 to 790 hours.

## Data Sharing Statement

The data that support the findings of this study are available from the corresponding author upon reasonable request.

## Conflicts of Interest

K.F. received grant/funding from Nobelpharma Co., Ltd. and CiCLE of the Japan Agency for Medical Research and Development. I.H., T.N., and T.W. are employees of Nobelpharma Co., Ltd. S.E. has received consultation fees from Nobelpharma Co., Ltd.

## Supporting information

Supporting information
Figure S1: Study design.
Figure S2: Participant disposition.
