# Supplementary material for: Pharmacokinetics, Safety, and Tolerability of NPC‐21, an Anti‐Cytomegalovirus Monoclonal Antibody, in Healthy Japanese and White Adult Men: A Randomized, Placebo‐Controlled, First‐in‐Human Phase 1 Study
Source: Clin Pharmacol Drug Dev. 2022 Jan 5;11(6):707–16. doi: 10.1002/cpdd.1065 (PMC9303920; doi:10.1002/cpdd.1065)

Furihata *et al.*, Pharmacokinetics, Safety, and Tolerability of NPC-21, an Anti-Cytomegalovirus Monoclonal Antibody, in Healthy Japanese and White Adult Men: A Randomized, Placebo-Controlled, First-in-Human Phase 1 Study

Supplementary Material:

Legends to Supplementary Figures:

Figure S1: Study design.

Figure S2: Participant disposition.

Supplementary Figure S1:

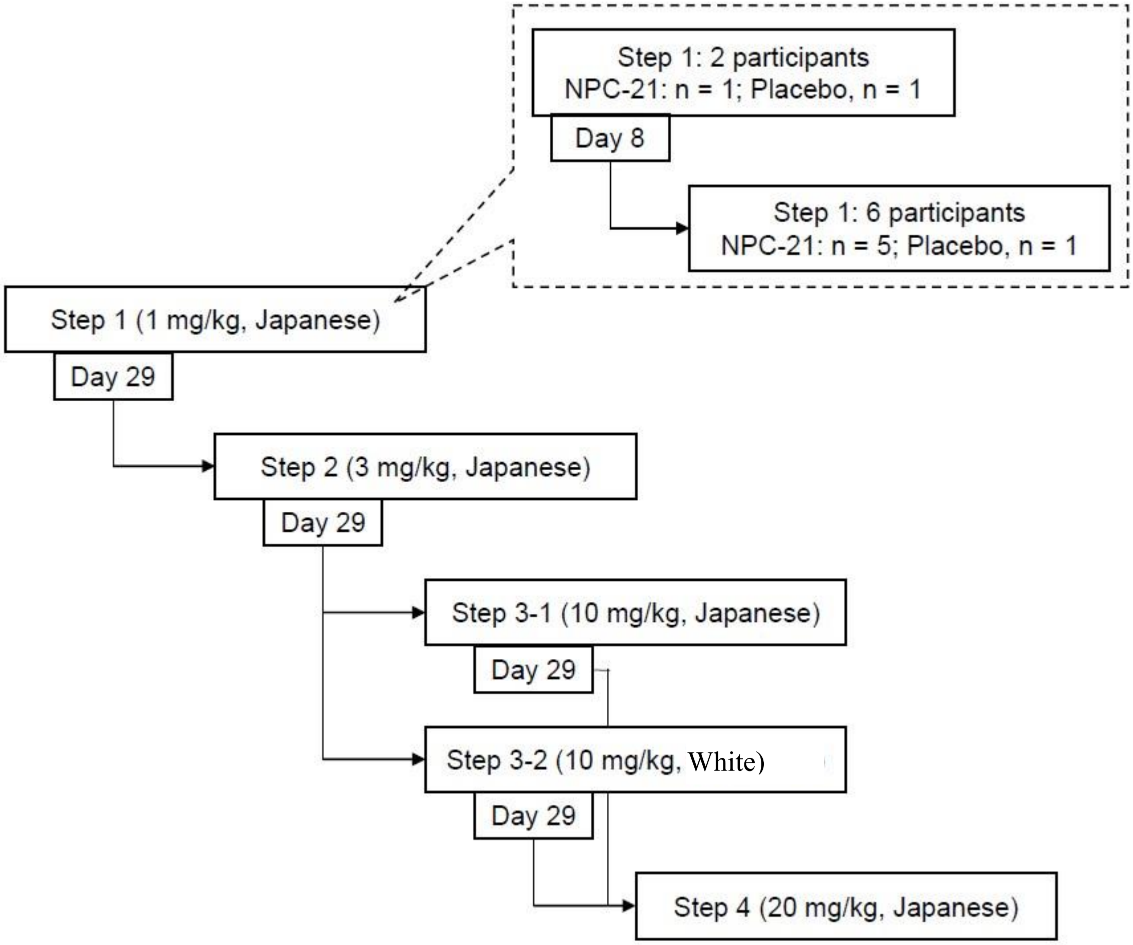

Supplementary Figure S2:

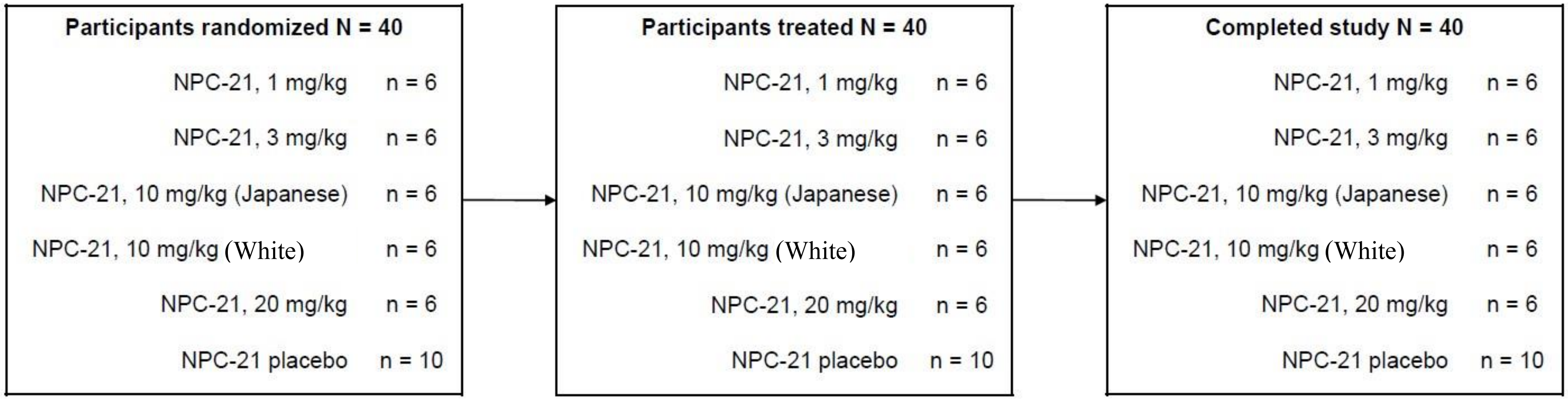

Supplement: Supplementary file 1 — Supporting information Figure S1: Study design. Figure S2: Participant disposition. [file CPDD-11-707-s001.pdf]
